# Lead-Free Potassium Sodium Niobate-Based Wearable Ultrasonic Patches for Blood Pressure Detection

**DOI:** 10.3390/mi16040392

**Published:** 2025-03-28

**Authors:** Yajun Sun, Yi Quan, Jie Xing, Zhi Tan, Xinhao Sun, Lifei Lou, Chunlong Fei, Jianguo Zhu, Yintang Yang

**Affiliations:** 1 Faculty of Intergrated Circuit, Xidian University, Xi’an 710071, China; 22111213644@stu.xidian.edu.cn (Y.S.); sunxinhao@xidian.edu.cn (X.S.); loulifei@mail.xidian.edu.cn (L.L.); clfei@xidian.edu.cn (C.F.); ytyang@xidian.edu.cn (Y.Y.); 2 College of Materials Science and Engineering, Sichuan University, Chengdu 610064, China; tanzhi0838@163.com (Z.T.); nic0400@scu.edu.cn (J.Z.)

**Keywords:** lead-free, KNN, ultrasonic transducers, wearable, blood pressure detection

## Abstract

Ultrasound is one of the most promising methods for blood pressure monitoring due to its harmless, non-invasive, and high-precision characteristics. To further enhance the biocompatibility of ultrasound blood pressure monitors, this work reports wearable ultrasonic patches for blood pressure monitoring based on lead-free KNN (potassium sodium niobate)-based materials. The patches are designed and fabricated with a center frequency of 5 MHz and dimensions of 2.8 mm × 2.8 mm, optimized for both electrical impedance matching and vascular detection. Moreover, biocompatible silicone rubber is used for the packaging. The wearable ultrasonic patches are demonstrated to effectively transmit and receive signals. The diameter of artificial blood vessels is measured to validate the vascular diameter detection capability of the patches. The relationship between blood pressure and vascular diameter is then calculated. A radial artery vascular system platform is built to simulate changes in human blood pressure. Finally, the patches are shown to successfully measure the variation in vessel diameters on this platform. These patches exhibit sufficient detection ability, good biocompatibility, and can adhere tightly to human skin without coupling agents, providing the possibility for safe, sustainable, comfortable, and wearable long-term blood pressure monitoring.

## 1. Introduction

According to the latest Global Hypertension Report [1], approximately one-third of adults worldwide are affected by hypertension. Originating from unhealthy lifestyles, prolonged sitting, staying up late, irregular eating habits, and anxiety, the number of hypertensive patients rapidly increased from 650 million in 1990 to 1.3 billion in 2019 [2]. Hypertension can lead to severe diseases such as stroke, heart attack, heart failure, and kidney damage, causing over 10 million deaths worldwide annually. Measuring blood pressure is a common method for diagnosing hypertension. Clinically, hypertension is defined as systolic blood pressure (SBP) beyond the normal range of 90–140 mmHg and diastolic blood pressure (DBP) beyond 60–90 mmHg [2]. Currently, blood pressure detection methods are divided into invasive and non-invasive approaches. Invasive blood pressure detection involves catheter insertion [3,4], where a catheter (based on fiber pressure sensors) is inserted into the arterial wall to achieve accurate blood pressure measurements. However, this method requires a sterile environment and increases the risk of infection, making it suitable only for patients who are critically ill. Non-invasive blood pressure monitoring instruments [5,6,7,8,9] mainly include manual auscultatory sphygmomanometers and cuff or wrist electronic sphygmomanometers. Most mercury and electronic sphygmomanometers are of the cuff type and require pressure application and release, making continuous long-term monitoring difficult.

Therefore, there is an urgent need for a non-invasive, continuous, and convenient blood pressure monitoring method. The ultrasonic blood pressure detection method [10] is widely used in clinical medicine due to its high precision and non-invasive nature. Its integration with wearable electronics technology is a current research hotspot. For example, Feng Xue’s team at Tsinghua University [11] developed a wearable Doppler ultrasound device that utilizes the dual-beam Doppler principle to achieve a quantitative measurement of blood flow velocity. Sheng Xu et al. [12] designed an integrated wearable ultrasound patch (USoP) for the long-term monitoring of deep tissues in moving human targets. Zheng Hairong [13], in collaboration with Professor Zhu Xuefeng, developed a novel wearable acoustic metasurface functional device capable of high/ultra-resolution medical imaging and precise drug delivery. However, these devices utilize lead-containing piezoelectric materials, and their fabrication processes are relatively complex.

According to the EU Directive 2011/65/EU (RoHS Directive) [14], the use of specific hazardous substances, including lead, in electronics and electronic components is restricted. Common piezoelectric materials, such as PZT series, contain lead components and are unsuitable for medical applications due to strong regulatory limitations. In the medical testing field, devices must not only meet safety, environmental health, and regulatory requirements but also ensure biocompatibility. According to the ISO-10993 [15] series standards, which are converted into the GB/T-16886 [16] series standards, the biological evaluation of medical devices is a crucial step in ensuring material biocompatibility. To achieve wearable patches suitable for long-term detection, biocompatible silicone rubber is experimentally determined as the packaging material.

In our previous work, we introduced a lead-free Cr-modified potassium sodium niobate-based (KNN-Cr) material [17] with the formula 0.97(K_0.45_Na_0.55_)Nb_0.955_Sb_0.045_O_3_-0.03(Bi_0.5_Na_0.5_)_0.9_(Li_0.5_Ce_0.5_)_0.1_HfO_3_-0.3%Fe_2_O_3_. This material is environmentally friendly and safe for humans and exhibits a high dielectric constant and superior performance compared to commercial piezoelectric materials such as PZT, facilitating miniaturization. Building on this foundation, we have engineered the patch parameters and integrated wearable conductors for interconnections. The frequency is set at 5 MHz to ensure sufficient detection depth and accuracy. Furthermore, we have utilized a biocompatible and pliant encapsulation that fits non-flat skin surfaces [18,19,20,21,22] without the need for coupling agents, enabling blood pressure detection.

## 2. Materials and Methods

To validate the performance of the patches, a comparison was made between the KNN-Cr-based piezoelectric ceramics used in this study and conventional PZT-5H piezoelectric materials (Table 1). The data for PZT-5H were sourced from he-shuai.com. The dielectric constant of the KNN-Cr-based piezoelectric ceramic was 3639, nearly three times higher than that of PZT-5H, offering higher performance and facilitating miniaturization. The *k*_t_ and *d*_33_ values were slightly lower than those of PZT-5H, while the *Z* value remained consistent, with minimal impact on the overall patch performance. The use of lead-free materials replaced lead-based materials, ensuring non-toxicity and environmental friendliness.

The main arteries in the human body include the brachial artery, the radial artery, the ulnar artery, and the carotid artery [23,24]. For measurement convenience, the radial and carotid arteries are primarily targeted [25]. This study focused on monitoring blood pressure in the radial artery, located on the outer side of the wrist, with a subcutaneous depth of 6–8 mm. Based on this depth, monitoring sensitivity, and spatial resolution requirements, a center frequency of 5 MHz was chosen. Using simulation software PiezoCAD, the appropriate design parameters were calculated with the equivalent circuit model (Supplementary Figure S1). The design parameters are shown in Table 2, with a piezoelectric layer thickness of 0.47 mm, a matching layer thickness of 0.096 mm, and a backing layer thickness of 1 mm. The average diameter of the radial artery is 2.3 mm ± 0.4 mm. Based on average diameter requirements and considering 50-ohm impedance matching, the patches size was determined to be 2.8 mm × 2.8 mm.

**Figure 1:**
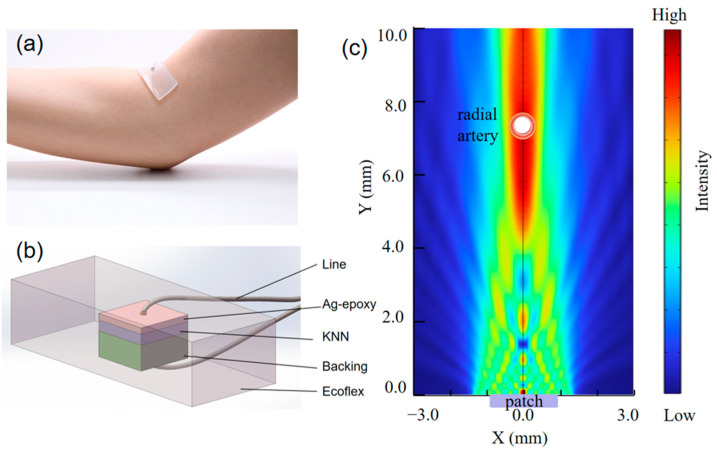
(**a**) Photograph of ultrasonic wearable patch adhering to skin for measuring the artery; (**b**) ultrasonic wearable patch structure; and (**c**) simulated sound field of patch at 5 MHz.

Figure 1a shows a patch adhered to the skin surface above the radial artery for wearable blood pressure monitoring. Figure 1b is a structural diagram for the finite-element analysis (FEA) simulation of the patch, with the middle purple part representing the KNN piezoelectric material layer; the upper pink part representing the matching layer, which reduces acoustic attenuation and enhances acoustic and electrical performance for better sound energy transmission; the lower green part representing the backing layer, which absorbs backward-radiated sound waves to reduce clutter interference; the gray-black part representing the solder paste, connecting the wires to the material for electrical signal transmission; and the outermost gray-transparent part representing biocompatible silicone rubber, serving as protection and ensuring wearability.

The FEA software COMSOL 6.1 was used to simulate the acoustic field of the patch, and the simulation results are shown in Figure 1c. The patch is placed at the 0 point on the X-axis, and the direction of the acoustic field is along the Y-axis. The radial artery is approximately located 6–8 mm beneath the skin. Taking the average, the acoustic field results are shown at a depth of 7 mm (Y = 7 mm) beneath the skin. The simulation results show that the acoustic field can completely cover the blood vessels at Y = 7 mm, indicating that the patches have sufficient detection accuracy and depth.

## 3. Results

### 3.1. Characterization of Ultrasonic Patches

**Figure 2:**
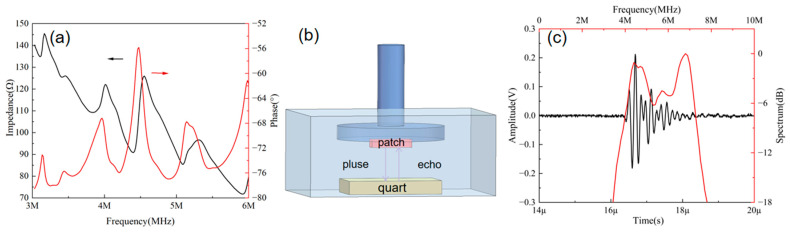
(**a**) Impedance phase results for the wearable patch. (**b**) Diagram of the pulse-echo testing environment. (**c**) Pulse-echo results for the wearable patch.

The electrical impedance of the ultrasonic patches was measured using an impedance analyzer (WK6500B 1J65120B, manufactured by Wayne Kerr Electronics, Bognor Regis, UK). Figure 2a shows the impedance phase results of the wearable patch, with the black line representing the impedance and the red line representing the phase. The X-axis corresponding to the peak of the red line denotes the central frequency, which aligns closely with the design and ensures adequate detection depth and accuracy. Figure 2b depicts the pulse-echo testing environment. The patch was adhered to a fixture with its emitting surface parallel to the underlying quartz block, submerged in deionized water. The quartz block served as the reflective element. The emitting surface of the patches was aligned parallel to the plane of the reflector. Using a pulse generator/receiver unit (model Olympus 5073PR, manufactured by Olympus, Waltham, MA, USA), the patches were individually actuated with pulsed waves. The fundamental parameters for the pulse generator/receiver were configured as follows: repetitive frequency 200 Hz, damping 30 Ω, energy 20 μJ, and gain of 0 dB. The output waveforms emitted by the pulse generator/receiver were captured by a 50 Ω coupled oscilloscope (model DSOX3024A, manufactured by Keysight, Santa Rosa, CA, USA), with the acquired waveforms subsequently stored for analysis. Figure 2c presents the results of the pulse-echo measurement, with a center frequency of 5.5 MHz, an echo amplitude of 394 mV, and −6 dB bandwidth of 65%. The black line represents the received echo signal, and the red line represents the bandwidth after FFT (Fast Fourier transform). These results indicate that the patches possess sufficient detection capability.

### 3.2. Principle of Blood Pressure Monitoring

**Figure 3:**
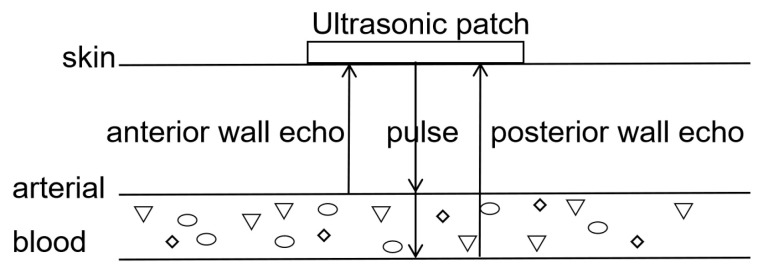
The diagram of the principle of blood pressure monitoring.

Human blood vessels are primarily composed of the adventitia, tunica media, tunica intima, and plasma [26]. For measurement convenience, the carotid or radial artery is generally chosen. Here, we propose a scheme for measuring radial artery blood pressure. The heart pumps blood to the arteries in a pulsating manner, and changes in blood flow velocity lead to changes in arterial diameter. The human cardiac cycle is about 1 s, during which blood is pushed from the heart towards the arteries [27]. The blood pressure inside the arteries increases, and the diameter expands to the systolic phase [28]. As blood flows back to the heart, the diameter of the arteries decreases to the diastolic phase. The principle of ultrasound blood pressure detection is shown in Figure 3.

During measurement, the patches are attached to the skin surface and emit ultrasound waves. When encountering the anterior wall of an arterial vessel, echo 1 is obtained at time *t*_1_. When encountering the posterior wall of the vessel, echo 2 is obtained at time *t*_2_. The diameter of the vessel is then determined using the following equation:

*D*(t) = *v* * (*t*_2_ − *t*_1_)/2. (1)

The arterial cross-sectional area is calculated as follows:

*A*(t) = pi * *D*^2^(t)/4. (2)

The blood pressure waveform is derived from the following:

*P*(t) = *p*_d_ * exp{*ɑ*[*A*(t)/*A*_d_ − 1]}, (3)

*ɑ* = *A*_d_In(*p*_s_/*p*_d_)/(*A*_s_ − *A*_d_). (4)

Here, *v* represents the speed of sound, *p*_d_ is the arterial diastolic pressure, *p*_s_ is the arterial systolic pressure, *A*_d_ is the arterial diastolic cross-sectional area, *A*_s_ is the arterial systolic cross-sectional area, and *α* is the stiffness coefficient of the blood vessels (which varies from person to person).

### 3.3. Biomimetic Vascular Testing Experiment

**Figure 4:**
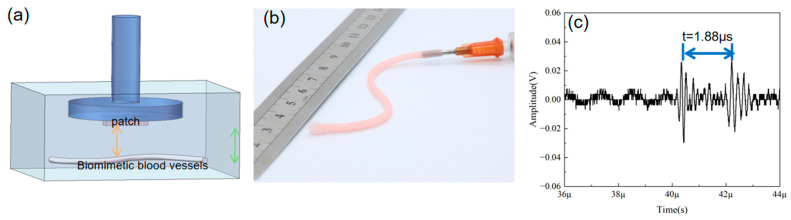
(**a**) Diagram of ultrasonic wearable patch measurement of biomimetic blood vessels. (**b**) Biomimetic vascular experimental patches. (**c**) Echo signals of biomimetic blood vessels.

To demonstrate the patches’ ability to accurately measure human blood vessel diameters, an artificial human blood vessel with a diameter of 1.4 mm was used as the target. The diagram of the artificial human blood vessel diameter measurement setup is shown in Figure 4a, with the blue part representing the clamping bracket to which the wearable patch is attached, facing the artificial blood vessel. The testing environment is similar to the pulse-echo experiment. The fundamental parameters for the pulse generator/receiver are configured as follows: repetitive frequency 200 Hz, damping 30 Ω, energy 20 μJ, and gain of 20 dB. The photograph of the artificial human blood vessel is shown in Figure 4b. The result is shown in Figure 4c, with a time interval of 1.88 µs between the two echo signals, yielding a calculated distance of 1.41 mm, consistent with the actual value. The amplitude of the measured echo 1 signal is 56 mV, and the amplitude of the echo 2 signal is 52 mV. These results indicate that the wearable patches can obtain the diameter size by calculating the echo time.

### 3.4. Construction and Testing of Bionic Vascular System

**Figure 5:**
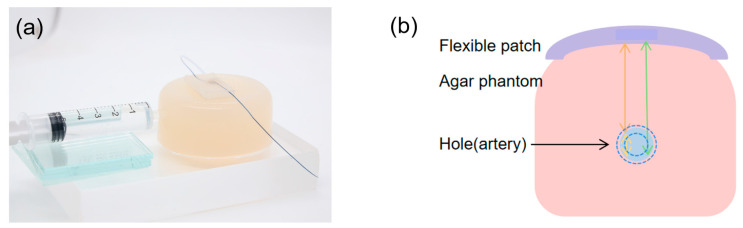
(**a**) Photograph of bionic vascular system. (**b**) Principle of blood vessel diameter testing.

To simulate the human radial artery, agar was used to create platforms. Agar powder was mixed with water in a ratio of 3:150, stirred and dissolved at 90 °C, and then cooled into the desired shape. A hole with a diameter of 2–3 mm was drilled at a depth of 6–8 mm from the surface of the agar block to simulate the radial artery. Water was injected into the hole using a syringe to simulate the changes in blood during diastole/systole, as shown in Figure 5a. Water was injected into the hole through a syringe, changing the aperture size to obtain a series of pulse-echo signals. The principle of measuring vessel diameter using the agar phantom is shown in Figure 5b. The wearable ultrasonic patch was placed directly above the hole in the agar block and connected to the SMA using a pulse emitter/receiver (Olympus 5073PR, Waltham, MA, USA). Subsequently, an oscilloscope (DSOX3024A, Keysight, Santa Rosa, CA, USA) was used to receive and store the received waveform.

**Figure 6:**
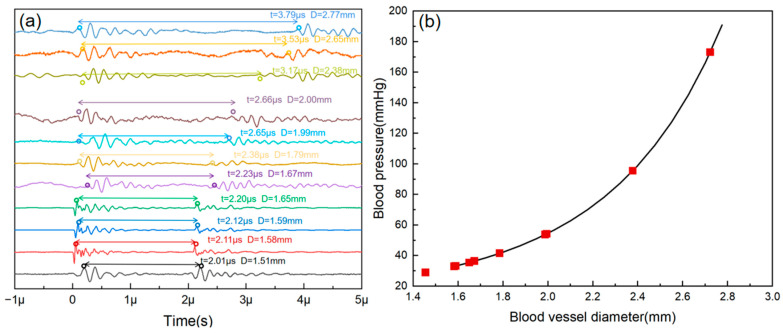
(**a**) Echo signals from simulated vascular phantoms with different aperture sizes tested using wearable patches. (**b**) Measured results (red dots) and the blood pressure–diameter relationship (black line).

The time intervals of the two echo signals are sorted from bottom to top in ascending order, as shown in Figure 6a, with values of *t* = 2.01 µs, 2.11 µs, 2.12 µs, 2.20 µs, 2.23 µs, 2.38 µs, 2.65 µs, 2.66 µs, 3.17 µs, 3.53 µs, and 3.79 µs. The corresponding diameters are D = 1.51 mm, 1.58 mm, 1.59 mm, 1.65 mm, 1.67 mm, 1.79 mm, 1.99 mm, 2.00 mm, 2.38 mm, 2.65 mm, and 2.77 mm, which are consistent with the actual measurements. For detailed information on each echo signal, please refer to Supplementary Material.

Based on previous research and empirical data, the average stiffness coefficient (α) is defined as 1.65 [24]. For healthy males, the diastolic pressure is 74.72 mmHg ± 8.61 mmHg, and the radial artery diameter is 2.39 mm ± 0.4 mm; for healthy females, the diastolic blood pressure is 72.48 mmHg ± 8.71 mmHg, and the radial artery diameter is 2.03 mm ± 0.38 mm. By averaging these values and substituting them into Equation (3), the relationship between radial artery diameter changes and blood pressure can be obtained, as shown by the black line in Figure 6b. The measured data points (red dots) indicate that the patches can achieve continuous and accurate detection of human blood pressure.

## 4. Discussion and Conclusions

This study has developed a flexible ultrasonic blood pressure monitoring patch based on lead-free piezoelectric materials, which complies with relevant medical regulations. Additionally, biocompatible silicone rubber is chosen for encapsulation, providing flexibility and a wearable fit to the human skin. The combination of ultrasound detection technology and lead-free KNN-Cr ceramic materials makes the patch non-invasive, non-toxic, and environmentally friendly. The central frequency of the flexible patch is 5 MHz, with dimensions of 2.8 mm × 2.8 mm, offering sufficient accuracy and depth for radial artery blood pressure monitoring. In pulse-echo experiments, the flexible patch demonstrated good detection capabilities and could measure the variation in vessel diameter in response to blood pressure variations.

To prove the accuracy of these patches in measuring the diameter of human blood vessels, an artificial blood vessel with a diameter of 1.4 mm was used as a target, and the results matched the actual values, indicating that the wearable patch can obtain the diameter size by calculating the time difference in echoes. Finally, an agar-based radial artery blood vessel platform was constructed, and water was injected into holes with a syringe to change the hole diameter, obtaining a series of pulse-echo signals. Based on empirical values and calculation formulas, the average values were substituted to obtain the relationship between radial artery diameter changes and blood pressure. Thus, it can be concluded that this ultrasonic flexible patch can achieve continuous and accurate blood pressure monitoring in the human body.

A −6 dB bandwidth is one of the most important ultrasonic device parameters for the accuracy of ultrasound detection, deciding the axial resolution. In this work, the patches achieved a −6 dB bandwidth value of 65%, which is at a high level for the studies utilizing ultrasound-based blood pressure measurement techniques. As a comparison, Peng et al. reported non-invasive and nonocclusive blood pressure monitoring via a flexible piezo-composite ultrasonic sensor, with a −6 dB bandwidth value of 46.93% [5]. Xu et al. reported a conformal ultrasonic device for monitoring central blood pressure, which has a −6 dB bandwidth value of about 30% [12].

This achievement has propelled the application of lead-free piezoelectric materials in the medical field and driven technological advancements in the field of ultrasonic flexible wearable devices, providing new possibilities for long-term, continuous, real-time monitoring of deep human tissues. This non-invasive, non-toxic, and environmentally friendly ultrasonic flexible blood pressure monitoring patch not only complies with relevant medical regulations but also ensures comfort and flexibility in wearing by using biocompatible silicone rubber as the encapsulation material. The development of these technologies indicates that wearable ultrasound devices will play a more significant role in health management, disease prevention, and clinical diagnosis in the future.

In addition, there is a lot of work that we should carry out in future studies before the patches can be used in clinic monitoring. The patches are operated by a big system. The miniaturization and wireless operation of the system are the most important issues that need to be addressed at present. Furthermore, although ecoflex rubber has excellent biocompatibility, it is not breathable. During long-term blood pressure monitoring, the accumulation of human sweat can cause the patch to adhere less tightly. Therefore, finding more suitable encapsulation materials for long-term human body adhesion is equally important.

## Figures and Tables

**Table 1:** Comparison of material parameters between KNN-Cr and PZT-5H.

	KNN-Cr	PZT-5H
Velocity (m/s)	5283	2890
Density (kg/m^3^)	4353	7900
Dielectric constant	3639	1390
*k*_t_	0.41	0.52
*Z* (MRayl)	23	23
*d*_33_ (pC/N)	465	680

**Table 2:** Design parameters of KNN-Cr ultrasonic wearable patch.

	KNN-Cr
Thickness of piezoelectric layer (mm)	0.47
Thickness of matching layer (mm)	0.096
Thickness of backing layer (mm)	1.00
Size of KNN-Cr (mm^2^)	2.8 × 2.8

## Data Availability

The original contributions presented in this study are included in the article/Supplementary Material. Further inquiries can be directed to the corresponding authors.

## Supplementary Materials

The following supporting information can be downloaded at https://www.mdpi.com/article/10.3390/mi16040392/s1: Figure S1: The pulse-echo simulations by equivalent circuit model; Figure S2: Results of vascular agar phantoms with different apertures.
