# Supplementary material for: Lead-Free Potassium Sodium Niobate-Based Wearable Ultrasonic Patches for Blood Pressure Detection
Source: Micromachines (Basel). 2025 Mar 28;16(4):392. doi: 10.3390/mi16040392 (PMC12029591; doi:10.3390/mi16040392)
Supplement: Supplementary file 1 [file micromachines-16-00392-s001.zip › micromachines-3520972-supplementary.pdf]

Supplementary Material

Lead-free KNN-based wearable ultransonic patches for blood pressure detection

Yajun Sun[1], Yi Quan[1,*], Jie Xing[2,*], Zhi Tan[2], Xinhao Sun[1], Lifei Lou[1], Chunlong Fei[1], Jianguo Zhu[2], Yintang Yang[1]

[1] Faculty of Intergrated Circuit, Xidian University, Xi'an 710071, China

[2] College of Materials Science and Engineering, Sichuan University, 610064, Chengdu, China

* Correspondence: quanyi@xidian.edu.cn; xingjie@scu.edu.cn;

According to equivalent circuit model, the simulated pulse-echo are shown in Supplementary Figure S1. Enter the initial piezoelectric parameters, set the target center frequency to 5 MHz, and achieve a better simulation target when the thickness of the piezoelectric layer is 0.47 mm, and the matching layer is Ag-Epoxy with a thickness of 0.096 mm.

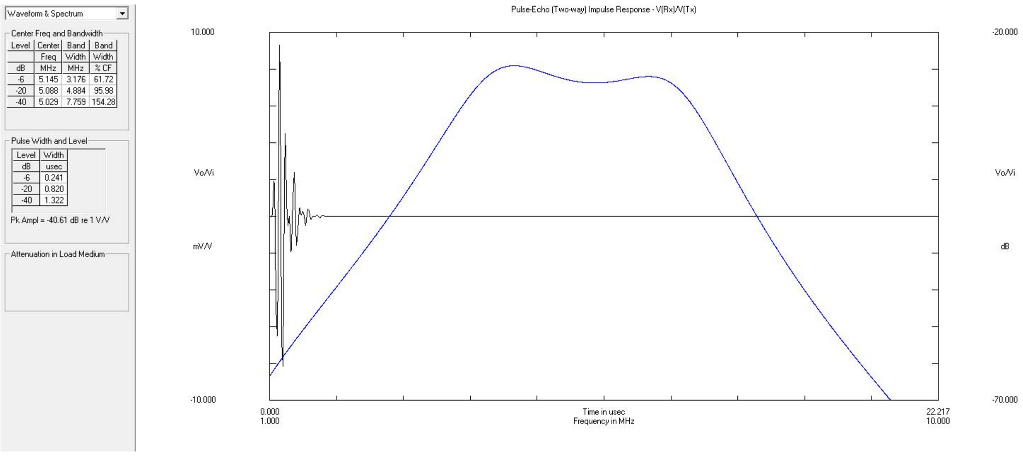

Figure S1. simulated results of pulse-echo.

Inject water into the hole through a syringe, change the aperture size, and obtain a series of pulse echo signals, as shown in Figure S2 (a)-(k), which are t=1.94 μs, t=2.11 μs, t=2.12 μs, t=2.2 μs, t=2.23 μs, t=2.38 μs, t=2.65 μs, t=2.66 μs, t=3.17 μs, t=3.63 μs, t=3.82 μs. The corresponding diameters are D=1.46 mm, D=1.58 mm, D=1.59 mm, D=1.65 mm, D=1.67 mm, D=1.79 mm, D=1.99 mm, D=2.00 mm, D=2.38 mm, D=2.72 mm, D=2.87 mm.

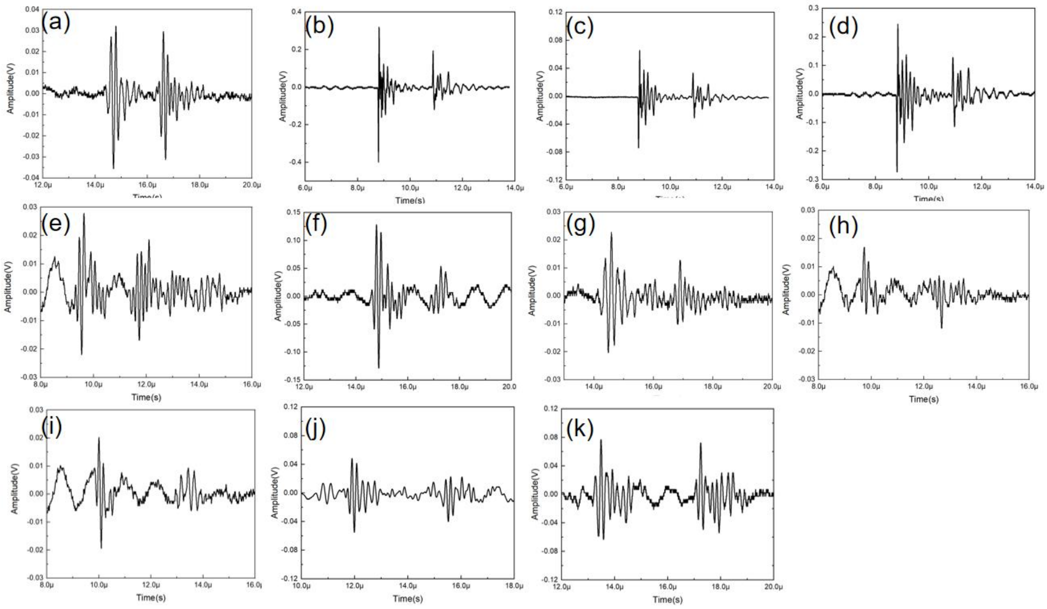

Figure S2. Results of vascular agar phantom with different apertures, from small to large.
